# Impact of the COVID‐19 pandemic on the treatment of acute cholangitis caused by choledocholithiasis: A single‐center retrospective study in Japan

**DOI:** 10.1002/deo2.371

**Published:** 2024-04-30

**Authors:** Jun‐ichi Hanatani, Koh Kitagawa, Fumimasa Tomooka, Shohei Asada, Akira Mitoro, Yukihisa Fujinaga, Norihisa Nishimura, Shinya Sato, Akihiko Shibamoto, Yuki Fujimoto, Takahiro Kubo, Satoshi Iwai, Yuki Tsuji, Tadashi Namisaki, Takemi Akahane, Kosuke Kaji, Misako Tanaka, Aritoshi Koizumi, Nobuyuki Yorioka, Takuya Matsuda, Hiroyuki Masuda, Masayoshi Takami, Mayuko Kikuchi, Mariya Kawanishi, Kazutaka Ohoka, Daisuke Watanabe, Akane Kawasaki, Hitoshi Yoshiji

**Affiliations:** ^1^ Department of Gastroenterology Nara Medical University Nara Japan; ^2^ Division of Endoscopy Nara Medical University Nara Japan

**Keywords:** endoscopic retrograde cholangiopancreatography, choledocholithiasis, COVID‐19, state of emergency, pandemic

## Abstract

**Objectives:**

This study aimed to determine the impact of the coronavirus disease 2019 (COVID‐19) pandemic on the treatment of acute cholangitis caused by choledocholithiasis.

**Methods:**

The Japanese government declared a state of emergency in April 2020 due to the COVID‐19 pandemic. We retrospectively reviewed the medical records of 309 patients who underwent endoscopic retrograde cholangiopancreatography (ERCP) for acute cholangitis caused by choledocholithiasis between April 2017 and December 2022.

**Results:**

Patients were categorized into a pregroup (*n* = 134) and a postgroup (*n* = 175), depending on whether they were diagnosed before or after the state of emergency declaration. The total number of ERCP cases and the number of ERCP cases with endoscopic stone removals increased after the state of emergency declaration. Compared with the pregroup, the numbers of patients with performance status of 0–1 and surgically altered anatomy increased, whereas the numbers of patients taking oral antiplatelets or anticoagulants and those with cerebrovascular disease decreased in the postgroup. The number of single‐stage endoscopic stone removals increased and hospital stays were significantly shorter in the postgroup. No differences in adverse event rates were detected between the two groups.

**Conclusions:**

Although our hospital provides tertiary care, the number of patients with cholangitis in good general condition and no underlying disease increased after the state of emergency declaration. The COVID‐19 pandemic resulted in an increase in the number of single‐stage endoscopic treatments and shortened hospital stays for patients with acute cholangitis caused by choledocholithiasis. No safety issues with ERCP were detected, even during the pandemic.

## INTRODUCTION

The coronavirus disease 2019 (COVID‐19) is an infectious disease caused by the severe acute respiratory syndrome coronavirus 2, which was detected in November 2019 and declared a global pandemic in March 2020 by the World Health Organization.^1^ In Japan, a strict lockdown policy was not implemented, and the government declared a state of emergency for the first time in April 2020. Early during the pandemic, COVID‐19 had been classified as a category 2 infectious disease, similar to tuberculosis in Japan. Therefore, all COVID‐19‐positive patients initially required inpatient treatment at a hospital specializing in infectious diseases. Elective medical procedures, such as cancer screening, were withheld to conserve medical resources and reduce the risk of in‐hospital transmission of COVID‐19.^2^ However, considering the exponential increase in the number of patients, inpatient beds soon became scarce, prompting local governments to decide that COVID‐19 patients with mild or asymptomatic symptoms could receive care at home or in hotels. COVID‐19 was then lowered to a category 5 infectious disease in May 2023.

Endoscopic care was affected by the restrictions on normal medical services due to the overwhelming number of COVID‐19 patients requiring care.^3^ The number of upper and lower endoscopic examinations in Japan decreased after the state of emergency declaration.^4^ Additionally, the number of people undergoing cancer screening decreased by 30.5% and that of cancer diagnoses decreased by 9.2% in 2020 compared with 2019, according to a report by the Japan Cancer Society.^5^ However, urgent endoscopic retrograde cholangiopancreatography (ERCP) remains a crucial therapeutic intervention, particularly during the pandemic. Limited research has investigated the frequency of ERCPs performed for acute cholangitis before and after the onset of the pandemic.^6^

Acute cholangitis is a gastrointestinal emergency requiring prompt recognition and treatment. The diagnostic and severity evaluation criteria for cholangitis have been standardized across various evidence‐based guidelines.^7^ Biliary decompression via ERCP is recommended as the initial treatment for patients with acute cholangitis. The incidence of choledocholithiasis, the most common cause of acute cholangitis,^8^, ^9^ has recently increased due to the aging of the population.^10^ Although choledocholithiasis may be asymptomatic, it causes cholangitis, pancreatitis, and obstructive jaundice in many patients.^11^ This study aimed to determine the impact of the COVID‐19 pandemic on the treatment of acute cholangitis caused by choledocholithiasis.

## METHODS

### Study population

Choledocholithiasis is the most common cause of acute cholangitis. Although emergency ERCP in patients with acute cholangitis caused by malignant biliary obstruction requires only biliary drainage, ERCP for acute cholangitis caused by choledocholithiasis requires stone removal, whether single‐stage or not. Thus, considerable variability in the ERCP procedure has been reported depending on the cause of acute cholangitis, with the present study focusing on acute cholangitis caused by choledocholithiasis. This single‐center retrospective study was approved by the Nara Medical University Ethics Committee (#2701) and was performed in accordance with the Strengthening the Reporting of Observational Studies in Epidemiology (STROBE) Statement. The requirement for patients’ written consent to participate in the study was waived due to its retrospective design; instead, an opt‐out approach was employed.

**FIGURE 1:**
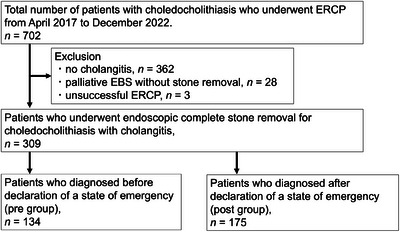
Flowchart of patient selection. (EBS, endoscopic biliary stenting; ERCP, endoscopic retrograde cholangiopancreatography).

Figure 1 shows the flow chart for patient inclusion in this study. We retrospectively reviewed the medical records of 702 patients who underwent ERCP for choledocholithiasis with cholangitis at Nara Medical University Hospital between April 2017 and December 2022. During the peak of the pandemic, the number of gastroenterology beds in our hospital was reduced by up to 40% from prepandemic levels, with beds being reallocated to COVID‐19 patients.

The diagnosis of choledocholithiasis was established through abdominal ultrasonography, computed tomography, or magnetic resonance cholangiopancreatography prior to ERCP. Patients were excluded from the study based on the following criteria: absence of acute cholangitis (*n* = 362), palliative endoscopic biliary stenting (EBS) without stone removal (*n* = 28), and unsuccessful ERCP (*n* = 3). Acute cholangitis was defined according to the diagnostic criteria outlined in the Tokyo Guidelines 2018. After excluding patients meeting the exclusion criteria, 309 patients who underwent complete stone removal for choledocholithiasis with cholangitis were included in the analysis. The decision to perform single‐stage stone removal or drainage only using EBS in the initial stage was determined by the physician based on the patient's condition and stone position. During the COVID‐19 pandemic, all patients with acute cholangitis underwent polymerase chain reaction testing for COVID‐19 prior to undergoing ERCP. None of the patients included in this study tested positive for COVID‐19.

### ERCP

To perform ERCP, patients were placed in the prone position and anesthetized/sedated with midazolam and buprenorphine hydrochloride or haloperidol and dexmedetomidine hydrochloride, as appropriate. Vital signs were monitored using an electrocardiogram. Oxygen saturation was assessed during the procedure, and patients were administered oxygen via nasal cannula when needed. A side‐viewing endoscope (JF‐260V, TJF‐260V; Olympus Medical) was used in the procedure. Alternatively, a balloon‐assisted enteroscope (EC‐450BI5; Fujifilm Medical) was used in patients with surgically altered anatomy (except for the Billroth I method). Selective bile duct cannulation was performed using a standard ERCP catheter (MTW ERCP catheter; MTW Endoskopie), with wire‐guided cannulation and contrast medium injection. Endoscopic sphincterotomies (ESTs) were performed using a sphincterotome (CleverCut 3V; Olympus Medical). A 6–10‐mm balloon catheter (HurricaneTM RX; Boston Scientific) was used for endoscopic papillary balloon dilation (EPBD), whereas a 12–20‐mm balloon catheter (CRE wire‐guided balloon dilator; Boston Scientific) was used for endoscopic large balloon dilation (EPLBD). CBD stones were removed via a basket (Medi‐Globe 8‐Wire Nitinol Basket; Medi‐Globe) and/or balloon catheter (Extraction balloon catheter, XEMEX, Tokyo, Japan) and/or mechanical lithotripter (XEMEX Lithotripsy Basket Catheter; XEMEX). Straight or pigtail plastic stents were used for EBS.

Although EST was prioritized for ampullary intervention, EPLBD was also used in cases wherein the diameter or number of stones was large. In cases wherein EST was difficult to perform, such as in those with large periampullary diverticulum or surgically altered anatomy, EPBD or EPLBD was occasionally performed without EST. No change in the endoscopic instruments or policy of ampullary intervention occurred during the study period, regardless of the pandemic.

The diagnosis and severity of cholangitis were defined according to the Tokyo Guidelines 2018.^7^ Adverse event (AE) severity was graded according to the American Society for Gastrointestinal Endoscopy lexicon.^12^

### Personal protective equipment

**FIGURE 2:**
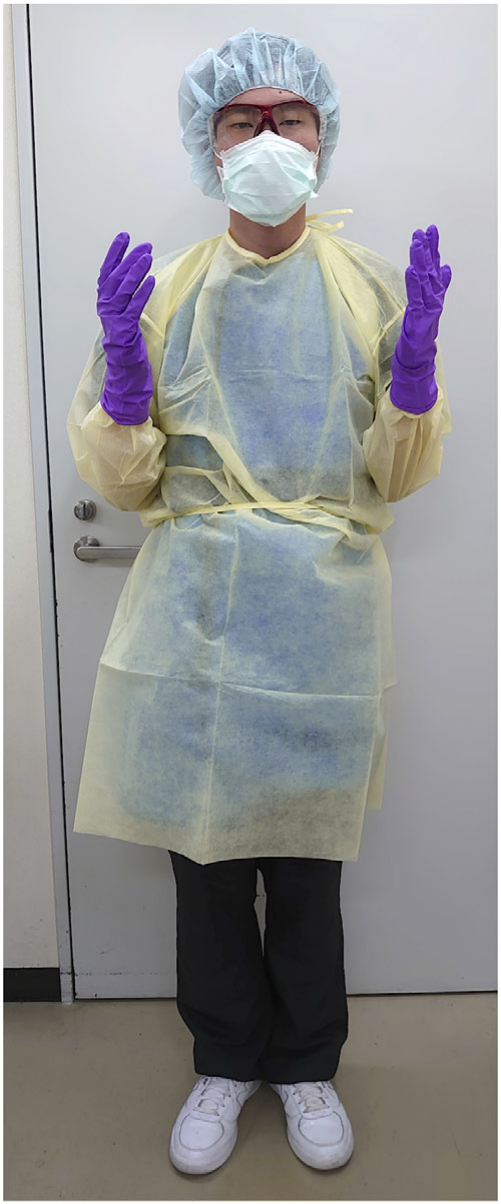
Personal protective equipment (PPE). During the pandemic period, we used this PPE in all routine upper gastrointestinal endoscopy procedures as well as endoscopic retrograde cholangiopancreatography.

Before the COVID‐19 pandemic, ERCPs were performed by endoscopists equipped with surgical masks, surgical gloves, and waterproof gowns. After the COVID‐19 pandemic, N95 masks, surgical haircaps, and eye protectors were also used. All doctors and nurses at our hospital received training on personal protective equipment (PPE) from the infection control team at the start of the pandemic. Even during routine upper gastrointestinal endoscopy, the doctors and nurses wore PPE in all cases (Figure 2).

### Statistical Analyses

Statistical analyses were performed using EZR ver. 1.41 (Saitama Medical Center, Jichi Medical University),^13^ which is a graphical user interface for R ver. 4.0.3 (The R Foundation for Statistical Computing). Categorical variables were compared using the chi‐squared test or Fisher's exact test. Continuous data with skewed distributions in the two groups were analyzed using a t‐test or the Mann–Whitney U test. Statistical significance was defined as a *p*‐value of <0.05.

## RESULTS

### Number of ERCP cases

**FIGURE 3:**
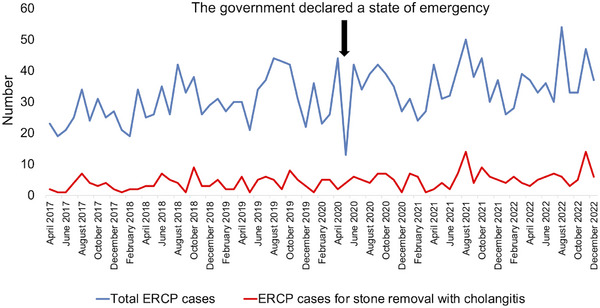
Number of ERCP cases and acute cholangitis ERCP cases with bile duct stone removal before (pregroup) and after (postgroup) the COVID‐19 pandemic. (COVID‐19, coronavirus disease 2019; ERCP, endoscopic retrograde cholangiopancreatography).

**FIGURE 4:**
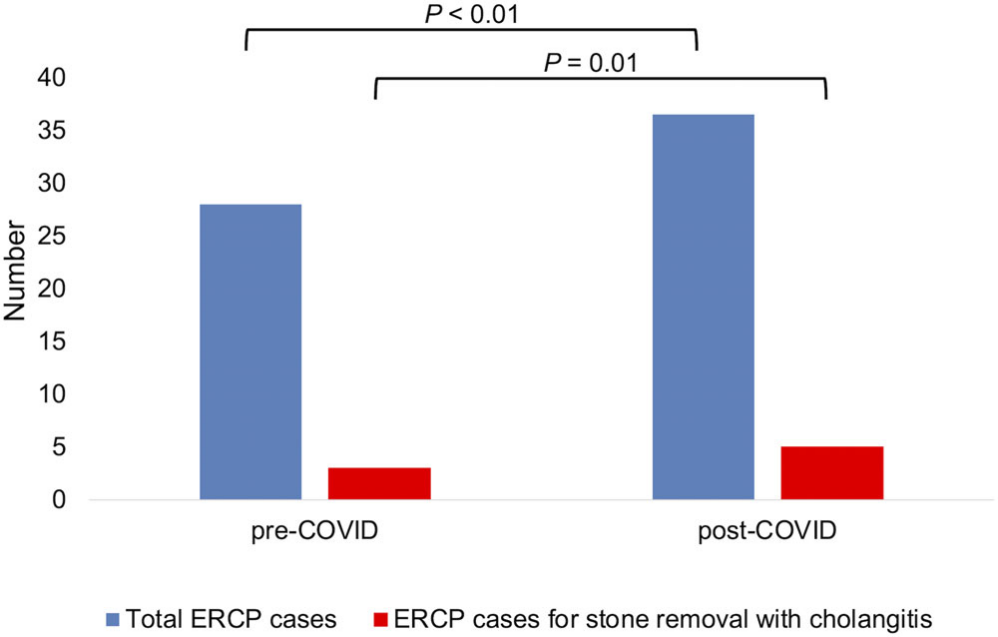
Comparison of the median number of ERCP cases per month before (pregroup) and after (postgroup) the COVID‐19 pandemic. (COVID‐19, coronavirus disease 2019; ERCP, endoscopic retrograde cholangiopancreatography).

Figure 3 shows the total number of ERCP cases and cases of acute cholangitis treated with bile duct stone removal before and after the COVID‐19 pandemic. The Japanese government declared a state of emergency due to the pandemic on April 16, 2020. In April and May 2020, the total number of ERCP cases decreased compared with previous months. However, after June 2020, the number of ERCP cases returned to pre‐emergency levels. The number of ERCP cases for treating acute cholangitis caused by choledocholithiasis remained unchanged; these cases were unaffected by the emergency declaration. The pregroup (before the emergency declaration) comprised 134 patients, whereas the postgroup (after the emergency declaration) comprised 175 patients. Figure 4 depicts the median number of ERCP cases per month before and after the COVID‐19 pandemic in the pregroup and postgroup. Both the overall number of ERCP cases and cases involving endoscopic stone removal increased after the emergency declaration.

### Baseline characteristics

Patient characteristics and demographic data on admission are presented in Table 1. A significantly higher proportion of patients in the pre‐group had received antiplatelet or anticoagulant therapy and had a history of cerebrovascular disease compared with the post‐group. Conversely, a significantly higher proportion of patients in the postgroup had World Health Organization performance status scores of 0–1, surgically altered anatomy, and periampullary diverticulum compared with the pregroup. No significant differences in age, sex, severity of cholangitis, cardiovascular disease, dementia, history of malignant neoplasm, number of stones, or diameter of stones were detected between the two groups.

**TABLE 1:** Patient characteristics.

	Pre‐group *n* = 134	Post‐group *n* = 175	*p‐*value
Age, median (range), years	76 (37–93)	75 (27–94)	0.73
Sex, male/female	89/45	120/55	0.71
Performance status score of 0–1, *n* (%)	123 (91.8)	174 (99.4)	<0.01
The severity of cholangitis, mild/moderate/severe	57/69/8	88/81/6	0.32
Antiplatelet or anticoagulant therapy, *n* (%)	38 (28.4)	29 (16.6)	0.02
Cardiovascular disease, *n* (%)	41 (30.6)	45 (25.7)	0.37
History of cerebrovascular disease, *n* (%)	19 (14.2)	12 (6.9)	0.04
Dementia, *n* (%)	9 (6.7)	5 (2.9)	0.73
History of malignant neoplasm, *n* (%)	31 (23.1)	55 (31.4)	0.12
Surgically altered anatomy, *n* (%)	7 (5.2)	25 (14.3)	0.01
Periampullary diverticulum, *n* (%)	32 (23.9)	63 (36.0)	0.02
Number of stones, median (range)	1 (1–10)	1 (1–10)	0.07
Diameter of stones, median (range), mm	6 (2–22)	7 (2–21)	0.85
Diameter of the common bile duct, median (range), mm	9 (4–25)	10 (4–25)	0.01

### Impact of the COVID‐19 pandemic on the treatment of patients with acute cholangitis caused by choledocholithiasis

Table 2 shows the endoscopic procedures. The number of single‐stage stone removals increased significantly in the postgroup compared with the pregroup (43.3%, 58/134 in the pregroup vs. 57.1%, 100/175 in the postgroup, *p* < 0.02). The proportion of patients who underwent EPBD was significantly higher in the pregroup compared with the postgroup. Conversely, the proportion of patients who underwent EPLBD alone without EST was significantly higher in the postgroup compared with the pregroup. No significant differences were detected in the portion of patients who underwent EST alone and EPLBD with EST. Hospital stays were significantly shorter in the post‐group compared with those in the pre‐group.

**TABLE 2:** Endoscopic procedure and adverse events.

	Pre‐group *n* = 134	Post‐group *n* = 175	*p‐*value
**Attempt of single‐stage stone removal in the first stage, *n* (%)**	58 (43.3)	100 (57.1)	<0.02
Success of complete stone removal in the first stage, *n* (%)	52 (90.0)	91 (91.0)	0.78
Procedure time in the first stage, median (range), minutes	21 (5–180)	21 (6–130)	0.91
Ampullary intervention			
EST alone, *n* (%)	70 (52.2)	81 (46.3)	0.30
EPBD, *n* (%)	32 (23.9)	19 (10.9)	<0.01
EPLBD alone, *n* (%)	11 (8.2)	39 (22.2)	<0.01
EPLBD with EST, *n* (%)	11 (8.2)	27 (15.4)	0.08
**Length of hospital stay, median (range), days**	14 (4–71)	10 (3–53)	<0.01
Adverse events, *n* (%)	7 (5.2)	16 (9.1)	0.27
Pancreatitis, *n* (%)	4 (3.0)	8 (4.6)	0.56
Bleeding, *n* (%)	1 (0.8)	7 (4.6)	0.14
Perforation, *n* (%)	1 (0.8)	1(0.6)	1.00
Aspiration pneumonia, *n* (%)	1(0.8)	0 (0)	0.43
Recurrence of stone, *n* (%)	15 (11.2)	16 (9.1)	0.57

Abbreviations: EPBD, Endoscopic papillary balloon dilation; EPLBD, endoscopic large balloon dilation; EST, endoscopic sphincterotomy.

Table 2 also shows AEs related to ERCP. No significant differences in AE rates were observed between the two groups. All patients who had pancreatitis exhibited improvements with conservative treatment. Bleeding occurred in one patient in the pregroup and seven patients in the postgroup. Hemostasis was achieved via endoscopic control without angiographic intervention or surgical operation in all patients who experienced bleeding. Perforation occurred in one patient in both the pregroup and postgroup; both patients exhibited improvements with conservative treatment without requiring surgical intervention. No procedure‐related deaths occurred.

## DISCUSSION

The number of ERCP cases increased, and the treatment approach for acute cholangitis caused by choledocholithiasis evolved during the COVID‐19 pandemic. Despite being a tertiary care institution, our hospital witnessed an increase in the number of patients with cholangitis arriving in good general condition as well as an increase in cases of single‐stage stone removal, leading to shorter hospital stays, during the COVID‐19 pandemic. Li et al. reported an increase in the proportion of patients with severe cholangitis during the COVID‐19 pandemic in Taiwan,^14^ which contrasts with our findings. However, in our medical area, no other hospitals were equipped to manage severe COVID‐19 cases and conduct emergency ERCP for patients with cholangitis. This likely resulted in the influx of fever and mild cholangitis cases to our hospital, which could have been managed elsewhere before the pandemic. With our hospital redirecting resources to accommodate COVID‐19 patients, including reducing gastroenterology beds by up to 40% compared with prepandemic levels, it became imperative to deliver treatment efficiently within shorter time frames, potentially contributing to the observed decrease in the length of hospital stays.

ERCP is a well‐established and indispensable technique for the treatment of acute cholangitis.^8^ Thus, we anticipated that the COVID‐19 pandemic would not reduce the number of urgent ERCPs. Table 3 presents studies documenting the impact of the COVID‐19 pandemic on ERCP procedures. Unlike upper endoscopies and colonoscopies, pancreaticobiliary disorders, such as bile duct obstruction or associated cholangitis, typically necessitate urgent intervention. Consistent with our findings, several studies have indicated that the number of ERCP cases did not decrease during the COVID‐19 pandemic.^14^, ^15^, ^16^ However, studies conducted in Italy, Korea, and Ireland reported a significant decline in the number of ERCPs due to the pandemic.^6^, ^17^, ^18^ Donato et al. noted a significant reduction in ERCP procedures during the COVID‐19 pandemic in Italy^17^ and concluded that this decrease could be attributed to reduced referrals from medium‐volume or primary care facilities and a decline in ER visits. Kim et al. reported a >60% decline in emergency ERCPs during the COVID‐19 pandemic in South Korea,^6^ linking this decline to reduced ER visits and referrals, as observed in Italy, as well as a decrease in the number of patients with acute pancreatitis and cholangitis caused by lifestyle changes prompted by lockdown policies. In our area, no tertiary care facilities other than our hospital could manage severe COVID‐19 cases; thus, referrals from other hospitals and ERs did not decrease. Moreover, the pandemic may have increased the number of emergency pancreaticobiliary cases. White et al. documented increased alcohol consumption and alcohol‐related deaths in the United States during the pandemic due to stress and stay‐at‐home measures.^19^ These studies highlight the variability in the impact of COVID‐19 on ERCP numbers across different countries, likely influenced by variations in medical policies and racial differences in the incidence of pancreaticobiliary diseases. Further large‐scale surveys are warranted to elucidate the underlying reasons for these differences.

**TABLE 3:** Summary of studies reporting the impact of the coronavirus disease 2019 (COVID‐19) pandemic on endoscopic retrograde cholangiopancreatography (ERCP) procedures.

Author (year)	Country	Single‐ or multicenter	Total number of ERCP cases	Procedure time	Procedure success rate	Rate of adverse events	Length of hospital stay
Donato et al.^17^ (2020)	Italy	Multi	Decreased	‐	‐	No difference	‐
Salerno et al.^15^ (2020)	Italy	Multi	No difference	‐	‐	‐	‐
O'Grady et al.^18^ (2020)	Ireland	Single	Decreased	‐	‐	‐	‐
Kim et al.^6^ (2021)	South Korea	Single	Decreased	‐	‐	‐	‐
Tag‐Adeen et al.^16^ (2021)	Egypt	Single	No difference	Short	No difference	No difference	No difference
Li et al.^14^ (2023)	Taiwan	Single	No difference	‐	No difference	No difference	Extended
Present study	Japan	Single	Increased	No difference	No difference	No difference	Shortened

Abbreviation: ERCP, endoscopic retrograde cholangiopancreatography.

The debate over whether to prioritize biliary decompression alone or complete stone removal in a single stage as the initial treatment for acute cholangitis caused by choledocholithiasis remains contentious. EST is necessary for bile duct stone removal, and the procedure typically requires a longer duration than decompression alone. One study found that additional stone removal after ES in a single stage led to an increased incidence of hemorrhagic AEs.^20^ However, according to the Japanese guidelines updated in 2017 (Tokyo Guidelines 2018), bile duct stone removal following ES in a single stage may be considered in patients with mild or moderate acute cholangitis.^21^ In our study, the number of single‐stage endoscopic cases increased, hospital stays were shorter, and AEs did not increase after the pandemic compared with before. Furthermore, during the COVID‐19 pandemic, endoscopists were required to wear N95 masks and eye protectors, but this did not prolong procedure times or reduce the success rate of endoscopic stone removal. No cases of viral transmission from patients to endoscopists were observed during the study period.

Interestingly, the number of cases of cholangitis in good general condition has increased in our hospital since the pandemic. Concurrently, there has been an increase in the number of patients with intractable choledocholithiasis and surgically altered anatomy. This may appear contradictory. However, patients with choledocholithiasis and surgically altered anatomy pose challenges for medium‐volume hospitals in terms of treatment. Thus, although there has been an increase in cases of cholangitis in good general condition, there has also been an increase in difficult‐to‐treat cases that can only be managed at tertiary care hospitals. Stone removal techniques, such as EPLBD for large and multiple stones, and balloon‐assisted enteroscopy ERCP (BAE‐ERCP), have recently been developed for patients with surgically altered anatomy.^22^, ^23^ However, BAE‐ERCP demands a high level of technical expertise.^23^ Despite the inclusion of a large number of difficult‐to‐treat cases in the study, patients with acute cholangitis caused by choledocholithiasis were treated safely and efficiently during the pandemic. Additionally, the post‐group underwent more EPLBDs, possibly due to the higher number of patients with choledocholithiasis and surgically altered anatomy.

This study has several limitations. First, this was a single‐center, retrospective study; thus, the generalizability of our results may be limited. A multicenter survey should be conducted to determine the applicability of our results to other areas. Second, the current study may have failed to reflect the true percentage of patients with severe cholangitis. Although we accepted as many patients who needed treatment as we could, we could not accept all of them due to the restrictions in normal medical care during the COVID‐19 pandemic. As such, more patients with severe cholangitis who could receive treatment at our hospital may have potentially existed. We also could not examine the duration between symptom onset and hospital visit. Thus, we cannot rule out the possibility that changes in the length of time patients waited at home during the pandemic affected the severity of cholangitis. Third, we could not determine the medical costs associated with polymerase chain reaction testing or PPE. Thus, it remains unclear whether shortening hospital stays in the postgroup was truly economically efficient.

In conclusion, the COVID‐19 pandemic resulted in an increase in the number of single‐stage endoscopic treatments and shortened hospital stays for patients with acute cholangitis caused by choledocholithiasis. However, no safety issues with ERCP were detected, even during the pandemic.

## CONFLICT OF INTEREST STATEMENT

None.
